# Light-Emitting Diode [LED]-Driven Mechanisms for Postharvest Decay Control and Functional Quality Improvement in Fruits and Vegetables

**DOI:** 10.3390/foods14172924

**Published:** 2025-08-22

**Authors:** Adejoke O. Obajuluwa, Dharini Sivakumar

**Affiliations:** 1 Phytochemical Food Network Research Group, Department of Crop Sciences, Faculty of Science, Tshwane University of Technology, Pretoria 0001, South Africa; obajuluwaao@tut.ac.za; 2 Queensland Alliance for Agriculture and Food Innovation, Centre for Food Science and Nutrition, The University of Queensland, Brisbane, QLD 4068, Australia

**Keywords:** Postharvest treatments, fruits, vegetables, fungal, decay, innate defense, functional, quality

## Abstract

Postharvest losses due to fungal decay pose a significant challenge to global fruit and vegetable production, especially in regions where rot pathogens are prevalent. Traditional control methods rely heavily on synthetic fungicides, which are increasingly criticized for their environmental risks, human health concerns, and their role in fostering pathogen resistance. These issues underscore the urgent need for sustainable, residue-free alternatives that not only manage postharvest diseases but also enhance produce quality. Light-emitting diode [LED] technology has emerged as a promising, eco-friendly solution capable of modulating plant physiological responses through specific light wavelengths. However, the exact defense mechanisms activated by LED exposure in postharvest decay control and nutritional enhancement remain underexplored. This review provides a comprehensive synthesis of recent findings on LED-induced control of fungal decay, focusing on how LED treatments modulate pathogen–fruit interactions, activate innate defense pathways, regulate gene networks linked to defense and nutritional traits, and contribute to improved fruit and vegetable quality and health benefits.

## 1. Introduction

Fruits and vegetables are essential components of a balanced diet, valued for their rich composition of phytonutrients, vitamins, minerals, and dietary fiber, which contribute to numerous health benefits [1,2,3]. South Africa is a major exporter of agricultural commodities such as citrus, apples, pears, grapes, avocados, litchis, and a variety of vegetables, including tomatoes, potatoes, and peppers [4]. To meet the stringent safety and quality standards of export markets in the European Union and beyond, stakeholders strive to improve supply chain efficiency and maintain produce integrity [5,6,7]. However, fungal spoilage during postharvest storage remains a critical bottleneck, significantly affecting produce quality, shelf life, and marketability, while contributing to major economic losses [8,9,10]. This inflicts major economic losses on fruit crops, affecting their quality and leading to negative consequences for export and marketability [11,12,13]. Fruit and vegetable pathogens such as *Colletotrichum* spp., *Botrytis cinerea*, *Monilinia* spp., *Penicillium* spp., *Rhizopus* spp., and *Fusarium* spp. infect a wide range of crops, [14,15,16], often causing latent infections that manifest during transportation or storage [17,18,19]. These pathogens exhibit high genetic variability and broad host ranges, complicating the development of durable control strategies [20,21], while efforts to breed resistant cultivars are constrained by the polygenic nature of resistance traits and a limited genetic pool [22,23,24]. Although chemical fungicides remain the primary intervention, their long-term sustainability is questionable due to environmental and health risks, pathogen adaptability, regulatory restrictions, and reduced efficacy against latent infections [18,25,26,27]. Consequently, there is increasing demand for eco-friendly technologies that can manage postharvest diseases while preserving fruit quality. Recent studies have highlighted the potential of LED technology to suppress fungal pathogens and enhance produce quality through spectral manipulation [28,29,30,31,32]. LEDs offer several advantages, including energy efficiency, wavelength specificity, and minimal heat generation [33,34]. Specific wavelengths—particularly blue, red, green, and UV-A—can stimulate innate defense responses, inhibit fungal growth, regulate gene expression, and elevate phenolic content [29,30,35]. Nevertheless, the molecular basis of LED-induced physiological changes, especially those involved in postharvest pathogen resistance and nutritional enhancement, remains underexplored. This review aims to consolidate current insights on LED technology in postharvest rot management and quality improvement, with an emphasis on its biochemical and gene regulatory effects in fruits and vegetables.

## 2. LED Characteristics, Materials, and Wavelengths

Light-emitting diodes [LEDs] are solid-state devices that produce light through electroluminescence, converting electrical energy directly into photons. They are categorized by their peak emission wavelengths or colors, spanning a broad spectrum including ultraviolet [UV-C: 200–280 nm, UV-B: 280–320 nm, and UV-A: 320–400 nm] and visible light ranges such as violet [400–450 nm], blue [450–500 nm], green [500–570 nm], yellow [570–590 nm], orange [590–610 nm], red [610–670 nm], and extending into near-infrared and far-red [700–1000 nm] regions [36,37,38,39]. LEDs emit narrow-bandwidth light by allowing current to flow through a p–n junction in semiconducting materials, enabling precise tuning of wavelength outputs to induce targeted biological responses [40,41]. The semiconductor materials used—often incorporating gallium, indium, silicon, nitrides, or synthetic sapphire—are doped with specific impurities to create desired electrical and optical properties. These material combinations influence the LED’s spectral characteristics, light intensity, and efficiency [42,43]. A typical LED system comprises several key components: the semiconductor chip, an optical encapsulant or lens, a heat sink, and a power supply. The encapsulant helps direct the emitted light efficiently to the target surface—such as fruit exteriors—while heat sinks manage thermal output, ensuring consistent performance and longevity of the device. These features make LEDs especially suited for postharvest applications, allowing precise delivery of specific light wavelengths and intensities that can trigger beneficial physiological responses in fruits and vegetables [44,45,46].

## 3. Rot Pathogenicity and Evasion of Defense Mechanisms in Fruits and Vegetables

**Figure 1:**
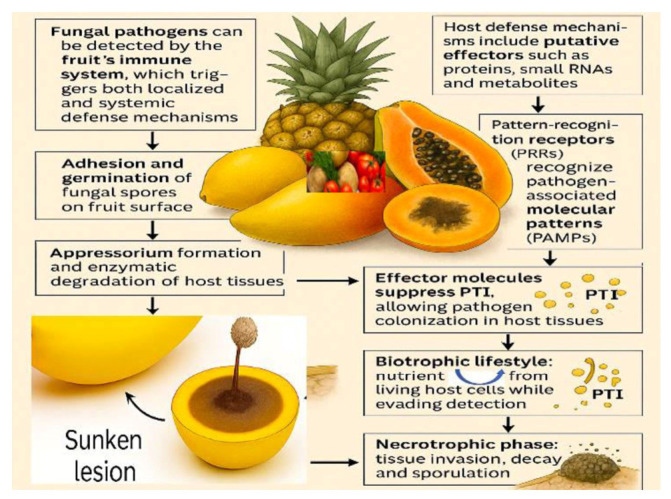
A Schematic diagram representing fungal rot pathogenicity and evasion of defense mechanisms in fruits and vegetables.

Postharvest rot caused by fungal pathogens is a major concern in horticultural crops, significantly affecting the quality and marketability of fruits and vegetables such as tomatoes, potatoes, mangoes, papayas, avocados, and guavas [11,25]. Pathogens like *Botrytis cinerea*, *Alternaria* spp., *Penicillium* spp., *Rhizopus* spp., and *Aspergillus* spp. are characterized by their high genetic variability and diverse infection strategies, both between and within populations [9,23,47,48]. Despite their varied pathogenic mechanisms, these fungi are generally detected by the plant immune system, which initiates both localized and systemic responses [49,50]. A common trait among these pathogens is their capacity to remain latent during fruit or vegetable development, only becoming active during ripening and storage, where they degrade tissues and reduce nutrient quality. Fungal infection typically begins with spore attachment and germination on the plant surface, followed by the formation of specialized structures like appressoria and the secretion of enzymes that degrade the host cell walls [19,51]. To facilitate host colonization, these pathogens deploy a variety of virulence factors, including proteins, small RNAs, and secondary metabolites that suppress host defenses [52,53]. The first line of plant defense involves pattern recognition receptors [PRRs] that detect pathogen-associated molecular patterns [PAMPs], activating PAMP-triggered immunity [PTI]. However, many fungi can release effectors that block PTI and allow further invasion [54]. Once inside the host, fungi may adopt a biotrophic lifestyle, extracting nutrients from living cells while avoiding immune detection (Figure 1). As host tissues senesce and immunity wanes, pathogens shift to necrotrophic behavior, resulting in visible symptoms such as sunken lesions, discoloration, and spore formation [55,56]. The fruit or vegetable’s ability to resist infection depends on both physical and biochemical barriers. The outer cuticle serves as the first line of defense, limiting water accumulation and fungal adhesion. Its thickness, composition, and structural integrity—especially the presence of cutin and waxes—play a crucial role in preventing pathogen ingress by reducing surface moisture, which is essential for spore germination [57,58].

## 4. LED Control of Fruit Rot-Related Pathogens in Fruits

In living systems, light influences processes such as growth, morphogenesis, secondary metabolism, and circadian rhythm regulation [59,60,61,62] and is a key regulator of fungal physiology [63,64,65]. Transcriptomic analyses have revealed that light exposure can differentially regulate over 5926 fungal genes, underscoring its significant role in fungal development and behavior [66]. Recent studies have demonstrated the effectiveness of LED light treatments in controlling fruit rot caused by pathogenic fungi. For example, *Colletotrichum acutatum* growth on strawberries was significantly inhibited when exposed to blue [450 nm] and green [530 nm] LED light at 50 μmol m^−2^s^−1^ [67]. Similarly, exposing red dragon fruit peels to 450 nm blue light at 300 Lux for two hours reduced decay incidence from 86.22% in untreated fruits to just 15.23% [68]. Supplemental LED lighting in greenhouses has also proven effective. In strawberries infected with *Colletotrichum gloeosporioides*, red and blue LED treatments not only reduced disease severity but also improved plant growth, demonstrating the dual benefits of disease suppression and physiological enhancement [69]^.^ In another study, 385 nm of blue light applied at 250 mW cm^−2^ for 10 min or 125 mW cm^−2^ for 20 min significantly suppressed fungal growth in mandarins six days after treatment [70]. Further, blue LED light at 465 nm was shown to reduce *Penicillium italicum* sporulation in Satsuma mandarins at both high [80 μmol m^−2^ s^−1^] and low [8 μmol m^−2^ s^−1^] fluence intensities [71]. In avocados, red LED exposure reduced anthracnose incidence and upregulated defense-related genes, suggesting that red light enhances host resistance mechanisms [72]. This enhancement is often linked to increased synthesis of phenolic compounds, which possess antifungal and antioxidant properties [73,74,75]. A growing number of studies confirm that specific LED wavelengths can simultaneously suppress pathogen development [30,63] and activate host immune responses in postharvest fruits and vegetables. Table 1 summarizes recent applications of LED light in the control of major fungal pathogens in diverse fruit types.

## 5. Mechanism of LED Light Action in Triggering Innate Biochemical Defense Response in Fruits and Vegetables

**Figure 2:**
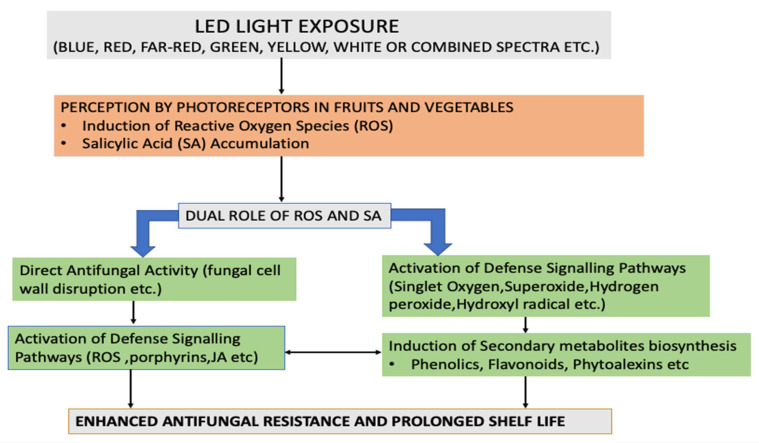
Mechanism of LED light’s action in triggering innate biochemical defense responses and induction of secondary metabolites in treated fruits and vegetables.

LED technology offers a sustainable and efficient solution for postharvest disease management due to its low energy consumption, minimal heat generation, and wavelength specificity. Blue LED light, in particular, has been shown to stimulate the accumulation of porphyrins and reactive oxygen species [ROS], which serve dual roles, directly inhibiting fungal pathogens and acting as intracellular signals to activate defense mechanisms in the host [80,81,82,83]. Elevated ROS levels can induce the expression of defense-related enzymes and compounds such as singlet oxygen, superoxide anions, hydrogen peroxide, and hydroxyl radicals [84,85,86]. These molecules contribute to the synthesis of secondary metabolites and enhance resistance against fungal invasion [87,88]. Unlike conventional fungicides, LED treatments are non-toxic and leave no chemical residues on fruit surfaces. Moreover, their spectral flexibility allows precise targeting of physiological pathways that promote the synthesis of phenolic compounds and pathogenesis-related [PR] proteins in host tissues [89,90,91]. Light exposure also promotes the biosynthesis of ROS and salicylic acid [SA], both of which are critical signaling molecules in plant immunity [92,93,94]. These responses are closely integrated with photomorphogenic pathways and plant hormones, such as jasmonic acid [JA], which regulate defense against fungal infections [95,96]. Salicylic acid [SA] plays a pivotal role in initiating local immune responses and inducing systemic acquired resistance [SAR], which offers long-lasting and broad-spectrum protection throughout the plant [97,98,99,100,101]. For example, red LED treatment in dragon fruit inoculated with *Colletotrichum* spp. helped maintain stable respiration and titratable acidity, while enhancing antioxidant activity and delaying senescence. This was attributed to the upregulation of metabolic and enzymatic defenses, as measured by DPPH radical-scavenging capacity [102]. These treatments also activate key enzymes such as phenylalanine ammonia-lyase [PAL], peroxidases [POD], chitinases, and other enzymes in the phenylpropanoid pathway—critical components of antifungal defense [103]. In strawberries, red and blue LED light has been found to reduce abiotic stress by increasing the activity of polyphenol oxidase and POD while also boosting anthocyanin accumulation [104]. LED exposure enhances the expression of PR proteins, reinforcing the fruit’s immune system. Red light, for instance, elevated levels of D-glucuronic acid—a precursor in the synthesis of pectin and hemicellulose—which plays a role in strengthening the cell wall and reducing susceptibility to fungal entry [105]. In purple capsicum, increasing the blue light proportion of the spectrum upregulated anthocyanin biosynthetic genes, leading to enhanced pigment accumulation [106]. In kiwifruit, blue light exposure delayed softening by downregulating ethylene biosynthesis, slowing starch degradation and preserving the integrity of the cell wall [107]. Similarly, red LED exposure in soybean seedlings increased levels of malonyl daidzin and genistin, while blue light boosted malonyl glycosides [108]. In wheat sprouts, blue LED exposure led to increased levels of gallic acid and quercetin—antioxidant and antimicrobial compounds—while decreasing p-coumaric acid and epicatechin, suggesting a rechanneling of metabolism towards stronger defense [109,110,111]. Several fungal pathogens are responsible for diseases including rots, molds, and spots, etc., accelerating spoilage and decay in fruits and vegetables [112,113], while phenolic compounds such as caffeic acid, vanillic acid, and epicatechin could inhibit fungal growth and stimulate the production of defense enzymes like chitinase and glucanase, which degrade fungal cell walls [114,115]. In avocados, caffeic and vanillic acid at 700 mg/L reduced mycelial growth, anthracnose incidence, and spore germination of *Colletotrichum gloeosporioides* in both in vitro and in vivo trials. These effects were attributed to enhanced activity of defense enzymes during storage [116]. Phenolic modulation through LED treatment also contributes to maintaining fruit firmness and extending shelf life [117,118,119]. The mechanism of LED light’s action in activating defense signaling pathways and inducing secondary metabolite production is shown in Figure 2.

## 6. Gene Expression Pathways Triggered by LED Light Exposure

LED light treatments influence gene expression pathways associated with light perception, hormonal signaling, and secondary metabolism, thereby enhancing fruit preservation, quality, and resistance to both biotic and abiotic stresses [36,43,120]. Transcriptomic studies reveal that LED exposure upregulates genes involved in stress response, antioxidant enzyme activity, cell wall reinforcement, and secondary metabolite production [121,122]. Genes encoding heat shock proteins, antioxidant enzymes—such as superoxide dismutase [SOD] and catalase [CAT]—and flavonoid biosynthetic enzymes [e.g., CHS, F3H] are commonly activated by LED irradiation. Blue light, in particular, regulates genes linked to circadian rhythm and photoreceptors, including transcription factors such as HY5 and PIFs, which orchestrate downstream light signaling networks [123]. In *Myrica rubra* [bayberry], red light treatment upregulated key anthocyanin biosynthesis genes such as *MrCHS*, *MrCHI*, *MrF3H*, *MrDFR1*, *MrANS*, and *MrUFGT*, as well as sugar metabolism-related genes including *MrSPS1*, *MrSPS2*, and *MrINV1*. The transcription factor *MrMYB1*, a key regulator of anthocyanin synthesis, was also activated, improving fruit coloration and sugar content [124]. In strawberries, red and blue LEDs increased yield and promoted the accumulation of phenolic compounds. Blue light primarily stimulated anthocyanin biosynthesis through upregulation of *FaC4H*, *FaCHS*, *FaF3H*, *FaDFR2, FaANS*, and the anthocyanin transport gene *FaRAP*. Red light, meanwhile, induced *FaCHS*, *FaCHI1*, and *FaUFGT1* expression [125,126,127]. In purple capsicum, exposure to 99% blue light [400–500 nm] at a photon flux density of 80 μmol m^−2^ s^−1^ over 28 days significantly upregulated anthocyanin biosynthesis genes, including *CaMYB*, *CaCHS*, *CaDFR*, *CaANS*, and *CaUFGT* [106]. Meanwhile, ethylene biosynthesis and ripening-related genes such as *NCED1*, *NCED2*, *NOR*, and *RIN* were downregulated, indicating a delay in senescence and respiratory activity [128]. Blue LED treatments are also linked to enhanced phenolic biosynthesis through activation of the phenylpropanoid pathway—an essential route for the production of flavonoids, lignins, and related compounds [109,129]. In chili peppers, blue and red LEDs stimulated the expression of carotenoid pathway genes [*Psy*, *Lcyb*, *CrtZ*, and *Ccs*], leading to increased carotenoid accumulation [130]. In strawberries, red light also enhanced resistance to *B. cinerea* via upregulation of the *FxaPE41* gene, which contributes to cell wall remodeling and defense [77]. In *Brassica rapa* [pak choi], LED treatment induced the expression of genes related to photosynthesis, glucosinolate biosynthesis, and chlorophyll retention, with 7761 genes showing differential expression in response to varied light spectra [122]. Similar responses were observed in apricot fruits, where white LED light increased expression of *LOX6* [lipoxygenase], *CEL* [endoglucanase family], and several peroxidase genes, while downregulating ripening-associated genes like *ACS*, *ACO*, and *HK* [121]. Table 2 provides detailed examples of how LED treatments influence gene expression across various fruit and vegetable crops, emphasizing the molecular basis of LED-mediated improvements in nutritional quality and disease resistance.

## 7. Phenolic Modulation for Improved Defense and Functional Value of Fruits and Vegetables with LED Light Treatments

LED Light-Induced Modulation for Enhanced Defense and Functional Quality of Fruits and Vegetables.

An increasing number of studies support the use of LED light treatments as an effective postharvest strategy to enhance fruit and vegetable quality through the modulation of phenolic compounds. Phenolics—including flavonoids, stilbenes, lignans, and tannins—are known for their strong antioxidant, anti-inflammatory, and anticancer properties, and are abundant in tropical and subtropical fruits [125,136,137,138,139]. LED light exposure stimulates the biosynthesis of polyphenols, flavonoids, and carotenoids—compounds that enhance both the antioxidant potential and the visual quality of produce. For instance, blue and red LED treatments have been shown to increase ascorbate and anthocyanin levels in mangoes and strawberries [104,140]. Ascorbate plays a vital role in plant development, stress resistance, and shelf-life extension [141,142,143], while elevated anthocyanin levels improve taste, color, and overall antioxidant capacity [144,145,146]. In strawberries, blue LED light induces anthocyanin accumulation via a signal transduction pathway involving the photoreceptor FaCRY1, the E3 ubiquitin ligase *FaCOP1*, and the transcription factor FaHY5. This *FaCRY1–FaCOP1–FaHY5* module is central to the plant’s response to blue light [125]. Red LED light alone has also been reported to promote the accumulation of phytochemicals in multiple crops [147,148]. LED lighting is particularly effective in enhancing the nutritional value of produce by regulating carotenoid biosynthesis. Red light exposure has been linked to increased levels of melatonin and carotenoids such as lycopene and β-carotene—compounds associated with human health benefits [107,149,150]. Conversely, inappropriate lighting can reduce the levels of antioxidant compounds [151], underscoring the need for spectral optimization. In leafy vegetables such as lettuce, primary antioxidants include ascorbic acid, carotenoids, and flavonoids, which play essential roles in mitigating oxidative stress by neutralizing free radicals [105,152,153,154]. In citrus fruits, blue LED light at 470 nm increased lutein content, reduced 9-cis-violaxanthin levels, and delayed senescence by enhancing chlorophyll retention in Valencia oranges [118]. Additional applications of 462 nm blue light every 10 days for 30 days raised vitamin C and total phenolic content in orange juice by 30% and improved the antioxidant profile of the peel [155]. Broad-spectrum white light [410–700 nm] also improved the nutritional quality and shelf life of mandarin oranges by increasing flavonoids such as quercetin rutinoside, chlorogenic acid, sinensetin, rutin, and naringin [156]. In blueberries, red [660 nm], blue [460 nm], yellow [590 nm], and white [380–800 nm] LEDs elevated levels of anthocyanins, ascorbic acid, glutathione, and total phenolics, while improving fruit size and membrane stability [131]. In tomatoes, diverse LED wavelengths [blue, green, white, red, and far-red] accelerated the accumulation of carotenoids, flavonoids, tocopherols, and phenolic acids, enhancing color development, antioxidant content, and postharvest performance [157]. Similarly, bananas treated with blue, green, and red LED lights for eight days showed improved peel coloration, ripening, ascorbic acid accumulation, and total phenolics [158]. Vegetables such as pak choi and broccoli also benefit from LED exposure. In pak choi, white light stimulated the accumulation of vitamin C and chlorophyll, while red and white LED combinations enhanced levels of polyphenols, flavonoids, glucosinolates, soluble sugars, and antioxidant activity [122,159]. Broccoli sprouts showed higher phenolic and glucosinolate content under white and yellow LEDs, contributing to shelf life and nutritional improvements [160]. Red LED treatment for five days also improved broccoli chlorophyll content and sensory quality [133]. Furthermore, white LEDs of varying intensities [3.6, 7.5, and 19.0 W m^−2^] promoted carotenoid biosynthesis while limiting ascorbic acid degradation in broccoli, extending shelf life and enhancing antioxidant profiles [161]. These findings underscore the promise of LED technology in boosting the nutritional value, visual appeal, and functional qualities of fruits and vegetables through targeted postharvest treatment strategies [Table 3].

## 8. Conclusions

LED light technology offers a sustainable, residue-free approach for postharvest management of fruit and vegetable decay, while simultaneously enhancing the nutritional functional quality. Through specific wavelength applications and spectral tuning, LED lights can activate plant defense pathways, suppress fungal pathogens, modulate gene expression related to ripening and senescence, and stimulate the biosynthesis of phenolic and antioxidant compounds. The adoption of LED technology aligns well with sustainable agricultural practices by reducing postharvest losses, minimizing chemical inputs, improving food quality and safety, and contributing to broader sustainability targets. To advance its adoption, future research should prioritize the development of optimized, crop-specific treatment protocols and further explore the molecular underpinnings of LED-induced responses. Evaluating scalability and integration into commercial postharvest infrastructure will also be essential for transitioning this promising technology from experimental to industry-wide practice.

## 9. Future Directions

While LED lighting—especially blue wavelengths—has demonstrated potential in inducing plant defense responses and stimulating the synthesis of protective compounds such as antioxidants and antimicrobial metabolites, further research is essential to deepen understanding of these effects. Specifically, there is a need to explore how different spectral combinations and light intensities modulate molecular and cellular defense mechanisms in fruits and vegetables. Future studies should focus on elucidating the roles of photoreceptors and signal transduction cascades and their interaction with the hormonal pathways involved in stress responses. Additionally, although beneficial under optimized conditions, excessive or inappropriate light exposure may be detrimental to plant tissues. Thus, it is crucial to establish standardized protocols for LED application, tailored to specific crop types, developmental stages, and postharvest contexts. As LED technology becomes increasingly integrated into commercial postharvest systems, a more refined understanding of plant–pathogen–light interactions will be vital for maximizing efficacy, reducing reliance on chemical fungicides, improving nutritive values, and achieving consistent, scalable outcomes. This knowledge will also contribute to the development of LED-based interventions that align with sustainability and food safety goals. Furthermore, interdisciplinary approaches that integrate omics technologies—such as transcriptomics, metabolomics, and proteomics—could uncover key regulatory networks and biomarkers associated with light-induced defense responses. Longitudinal studies assessing the residual effects of LED treatments during storage, distribution, and shelf life will also be instrumental in translating laboratory findings into real-world applications. Incorporating artificial intelligence and machine learning into the design of LED systems may provide precision control over treatment parameters, enabling real-time optimization for diverse storage conditions. Finally, socioeconomic and cost–benefit analyses are needed to evaluate the practical feasibility of adopting LED technologies across different scales of production and geographic regions.

## Figures and Tables

**Table 1:** Recent applications of LED against fungal pathogens of fruits and vegetables.

Fruit	Pathogen	LED Types and Treatment	Results/Observations	References
Strawberries	*Colletotrichum acutatum*	Blue [450 nm], green [530 nm], red [630 nm], far red [735 nm], and white [5700 k] at fluence rates of 50 μmol m^−2^ s^−1^, 100 μmol m^−2^ s^−1^, and 200 μmol m^−2^ s^−1^	High inhibition of *C. acutatum* under green, red, and blue LED lights	[67]
Strawberries	*Botrytis*. *cinerea*	White [300–800 nm], blue [460 nm], and red [660 nm] at a fluence rate of 10 µmol m^−2^ s^−1^	High inhibition of *B. cinerea* sporulation by red light, while blue, blue + red, and white lights inhibited sclerotia formation	[76]
Avocados	*Colletotrichum. gloeosporioides*	Blue and Red	Significantly lower anthracnose incidence [25%] in red LED light and [50%] in blue LED light	[72]
Late oranges	*Penicillium digitatum*	Blue [450 nm] at a fluence rate of 60 µmol m^−2^ s^−1^ for 2 days	Significantly reduced rot disease incidence [99–100%] and disease severity reduction [67–70%] in LED blue light-elicited fruits	[30]
Strawberries	*Botrytis cinerea*	Blue [450 nm], green [520 nm], and red [660 nm] at a fluence rate of 250 μmol m^−2 ^s^−1^ for 5 h/day	Lower AUDPC values [46.9 ± 8.4] at 36 h post-inoculation with red light	[77]
Strawberries	*Rhizopus stolonifer* and *Botrytis cinerea*	Violet [405 nm] at a fluence rate of 2.68 ± 0.5 mW/cm for 12 days	*R. stolonifer:* 3.4 CFU/g reduction *B. cinerea:* 1.9 log CFU/g reduction	[31]
Litchi	*Geotrichum candidum* and *Fusarium* sp.	Violet [410–420 nm], blue [460–470 nm], and green [520–530 nm] at fluence rates of 32.0 ± 0.15 W/m^2^, 49.2 ± 0.40 W/m^2^, and 60.4 ± 0.56 W/m^2^, respectively, scheduled at 2, 4, 6, 8, and 10 h illumination times	Reduced the population of *G. candidum* and *Fusarium* sp. by more than 2 log CFU/g [∼99%]	[32]
Nectarines	*Monilinia* spp. [*M. laxa*, *M. fructicola*, *M. fructigen*]	Blue [460 nm], red [660 nm], far-red [740 nm], UV-A [370 nm], and broad-spectrum white [400–700 nm]	*M. fructicola* growth rate was significantly reduced under red light wavelength	[29]
Strawberries	*Botrytis cinerea*, *Rhizopus stolonifer*	LED light [405 nm]	67% reduction of *B. cinerea* and 19% reduction of *R. stolonifer* population	[78]
Tomatoes	*Botrytis cinerea*, *Rhizopus stolonifer*	LED light [405 nm]	79% reduction of *B. cinerea* and 70% *R. stolonifer* population	[78]
Satsuma mandarins	*Penicillium italicum*	Blue LED [465 nm] at a fluence rate of 80 µmol m^−2^ s^−1^ [high] and 8 µmol m^−2^ s^−1^ [low]	Significant reduction of blue mold sporulation at both high and low fluence intensities	[71]
Cherry tomatoes	*Botrytis cinerea*	Purple [405 nm], blue [470 nm], green [530 nm], or red [660 nm] light at an intensity of 40 W m^−2^	Significant 17% and 12% gray mold incidence reduction in treated blue and green irradiated fruit compared to control	[28]
Citrus [Satsuma mandarins]	*Geotrichum. citri-aurantii*	Blue [455 nm] in varied photoperiods [negative control-darkness DD, 8 h light/16 h dark [8 LD], 16 h light/8 h dark [16 LD], and 24 h constant light [24 LL] at fluence rates of 50, 100, 150, and 200 μmol m^−2^ s ^−1^	Significant reduction of sour rot decay to 0%, 3.33% and 41.67% in *Citrus unshiu*, *Citrus sinensis* L. Osbeck, and *Citrus reticulata* Blanco cv. Ponkan, respectively, with blue light treatment at a fluence rate of 200 μmol m^−2^ s^−1^	[79]

**Table 2:** Recent evidence of gene expression regulation in fruits treated with LED lights.

Study	Fruit Type	LED Type and Exposure	Genes Involved in Phenolics Production	Enrichment Pathways
[118]	Valencia oranges	Blue [470 nm]	Upregulation of chlorophyll biosynthesis genes [*CitGGDR*, *CitCHLH*, *CitCHLM*, *CitCHL27*, *CitPORA*, and *CitCAO*]	Chlorophyll synthesis, color enhancement, and increased reactive species scavenging capacity
[125]	Strawberries	Red [660 nm] and blue [450 nm] for 96 h	Blue light upregulated anthocyanin biosynthetic enzyme genes [*FaC4H*, *FaCHS*, *FaF3H*, *FaDFR2*, *FaANS*] and anthocyanin transport gene [*FaRAP*], while red light upregulated *FaCHS, FaCHI1*, and *FaUFGT1*	Transcriptional chaperones of anthocyanin structural genes, signalling and synthesis, phenylpropanoid biosynthesis
[131]	Blueberries	Red [660 nm], blue [460 nm], yellow [590 nm], and white [380–800 nm]	Upregulation of anthocyanin biosynthesis genes—*VcC4H*, *Vc4CL*, *VcCHI*, *VcLDOX*, *VcDFR*, *VcUFGT*	Anthocyanin biosynthesis
[106]	Purple capsicum	Red [660 nm]	Upregulation of biosynthetic genes—*CaMYB*, *CaCHS*, *CaDFR*, *CaANS*, and *CaUFGT*	Anthocyanin biosynthesis
[121]	Ripe apricot fruits	White [450–460 nm] at a fluence of 5 μmol m^−2^ s^−1^ for 12 days	Upregulation of lipoxygenase [LOX 6], endoglucanase [CEL- *CEL6*, *CEL9*, *CEL10*, *CEL11*], peroxidase [POD—*PODA2*, *POD4*, *POD31*, *POD42*], while malate dehydrogenase [MDH], *1*-aminocyclopropane-1-carboxylate synthase [*ACS*], 1-aminocyclopropane-1-carboxylate oxidase [*ACO*], and hexokinase [*HK*] genes were downregulated	Ascorbate and aldarate metabolism, ethylene and flavonoid biosynthesis
[122]	Pak choi	White [448–549 nm], red [600–700 nm], green [500–599 nm], blue [400–499 nm], And far-red [701–780 nm] at fluence rates of 10 μmol m^−2^ s^−1^, 22.2 μmol m^−2^ s^−1^, 43.3 μmol m^−2^ s^−1^, 25.5 μmol m^−2^ s^−1^, and 2.3 μmol m^−2^ s^−1^, respectively	Distinct upregulation of *HemA*-related and chlorophyll synthesis genes—*chlI*, *chlE*, and *por* of the total of 2733 upregulated genes	Selenocompound metabolism, monoterpenoid biosynthesis, indole alkaloid biosynthesis, C5-branched dibasic acid metabolism, monobactam biosynthesis, glycosphingolipid biosynthesis, porphyrin and chlorophyll metabolism, nitrogen metabolism, amino sugar and nucleotide sugar metabolism, phenylalanine, tyrosine and tryptophan biosynthesis, circadian rhythm, carbon metabolism, ascorbate and aldarate metabolism, carbon fixation, amino acid biosynthesis, sulphur metabolism, glycosylate biosynthesis, glyoxylate and dicarboxylate metabolism, and photosynthesis
[119]	Pak choi	Red [65 μmol m^−2^ s^−1^], blue [50 μmol m^−2^ s^−1^], and red + blue [45 μmol m^−2^ s^−1^]	Upregulation of ethylene signaling gene [*BraEIN3*]	Ethylene biosynthesis
[132]	Pennywort	White LED [27 μmol m^−2^ s^−1^], dark, red LED [24.7 μmol m^−2 ^s^−1^ at 650 nm], blue LED [29.5 μmol m^−2^ s^−1^ at 450 nm], and green LED [30.5 μmol m^−2^ s^−1^ at 530 nm] for three days. In this study, white, red, blue, and green LEDs at an intensity range of 25–30 μmol m^−2^ s^−1^	Expression of triterpenoid biosynthesis genes, including *C. asiatica* squalene synthase [*CaSQS*], *C. asiatica* β-amyrin synthase [*CabAS*], and *C. asiatica UDP* gluclosyltransferase-73AH1 [*CaUGT73AH1*; *CaUGT*]	Triterpenoid biosynthesis
[133]	Broccoli	Red LED at a fluence rate of 50 μmol m^−2^ s^−1^	Suppression of chlorophyll degrading genes, chlorophyllase II [*BoCLH2*], chlorophyllase III [*BoCLH3*], and pheophorbide a oxygenase [*BoPAO*]	Porphyrin and chlorophyll metabolism
[134]	Pears	White LED [1200 lumens] at a fluence rate of 151 μmol/m^2^ s	Decreased relative expression of chlorophyll degradation-related genes *[PbASC4, PbACO1, PbETR1]* and increased expression of ethylene receptor genes *PbETR2*, *PbERS1*, and *PbERS2*	Chlorophyll metabolism and ethylene biosynthesis
[135]	Peppers [*Capsicum annuum* L.]	Red [700 nm], blue [465 nm], and full-spectrum white light, in different ratios at a fluence rate of 240 ± 30 µmol m^−2^ s^−1^	Increased expression of *ERF021*, *FAD2*, *ERF1B*, *ERF026*, *TM9SF7*, *ERF091*, *ERF012*, *TM9SF2*, and *ERF110* genes	Flavonoid [vitexin and cyanin] biosynthesis and ethylene-responsive factors
[28]	Cherry tomatoes	Purple [405 nm], blue [470 nm], green [530 nm], or red [660 nm] light at an intensity of 40 W m^−2^	Upregulated the genes encoding six defense-related enzymes, namely *LeCHI*, *LeGLU*, *LePAL*, *LeSOD*, *LePOD*, and *LeCAT*	PAL and secondary metabolite biosynthesis

**Table 3:** Studies of LED light-induced modulation for enhanced defense and functional quality of fruits and vegetables.

Fruit Type	LED Type and Exposure	Phenolic Compounds	Enhanced Fruit Qualities	Study Reference
Strawberries	Blue [460 nm], red [660 nm], and a combination of red and blue LEDs	Increased anthocyanin levels	Increased fruit mass, length, total chlorophyll, and total soluble solids. Improved potassium, iron, and magnesium levels	[162]
Valencia oranges	Blue [470 nm]	Increased lutein and decreased 9-*cis*-Violaxanthin	Two-times higher chlorophyll accumulation compared to non-treated orange fruits, enhanced color [regreening], and delayed senescence	[118]
Strawberries	Violet [405 nm] at 2.68 ± 0.5 mW/cm for 12 days	Significant increase in total phenolic content, anthocyanin content, and vitamin C content	Higher antioxidant levels and nutritive values	[31]
Red dragon fruit	Blue [450 nm] at 300 Lx for 2 h	Decreased ROS generation, reduced cell-wall monosaccharides, terpenes, and esters, and increased the activity of antioxidant enzymes	Improved fruit disease resistance and delayed fruit senescence by enhancing enzymatic antioxidant systems	[68]
Mandarin oranges	Broad-spectrum white [410–700 nm] at 150 ± 20 μmol photons m^−2^ s^−1^ for 7 days	Increased flavonoid quercetin rutinoside, chlorogenic acid, sinensetin, rutin, and naringin	Improved shelf life and nutritional quality of fruits	[156]
Dragon fruit	Red LED light [660 nm, 100 Lux for 24 h	Increased titratable acid [TA], total soluble solids [TSS], TSS-TA ratio, and DPPH scavenging potentials	Radically increased nutritive values and delayed fruit senescence	[102]
Strawberries	White, blue [450 nm], or red [730 nm] light during storage, stored for 16 h at a fluence of 100 μmolm^−2^ s^−1^ and 8 h of dark for 5 d	Modulation of anthocyanin and abscisic acid and regulation of auxin	Improved firmness, color, and taste	[140]
Strawberries	Red LED [660 nm] and blue [450 nm] for 96 h	Induced anthocyanin accumulation	Improved nutritive value, color, and taste	[125]
Pak choi	White [448 nm and 549 nm] at a fluence rate of 10 μmol m^−2^ s^−1^	Induced higher vitamin C and chlorophyll content	Improved shelf life and color	[122]
Blueberries	Red [660 nm], blue [ 460 nm], yellow [590 nm], and white [380–800 nm]	Accumulation of anthocyanin, higher total phenol content, including ascorbic acid and glutathione	Increased fruit width, height, and weight of blueberry fruits, enhanced cell membrane integrity resulting in improved firmness	[131]
Valencia oranges	Blue LED light [462 nm, at a fluence of 6.8 μmol m^−2^ s^−1^] every 10 days for a period of 30 days.	Increased vitamin C and total phenol contents increased by 30% in the orange juice	Increased total antioxidant capacity of the peel	[155]
Tomatoes	Blue [450 nm], green [520 nm], white, red [638 nm], and far-red [740 nm]	Fast accumulation of carotenoids, flavonoids, tocopherols, and phenolic acids; faster color development	Improved nutritive value and color and postharvest physiology	[157]
Bananas	Blue [464–474 nm], green [515–525 nm], and red [617–627 nm] for 8 days at fluence rates of 3920, 4340, and 5200 µmol photon m^−2^ s^−1^, respectively	Enhanced ethylene production, ascorbic acid, and total phenols	Ripening promotion, enhanced peel color, firmness, and taste	[158]
Broccoli sprouts	White [610 nm], yellow [600 nm], and green [517 nm] at a fluence rate of 35 ± 2.5 μmol m^−2^ s^−1^	Increased total phenolic content and total glucosinolate content under yellow and white LED lighting	Increased nutritive value and extended shelf life	[160]
Broccoli	Red [50 μmol m^−2^ s^−1^] for 5 days	Chlorophyll content modulation	Enhanced taste, higher sensory score, color, and weight	[133]
Red chard [*Beta vulgaris*]	Red [660 nm], green [ 517 nm], yellow [600 nm], white [610 nm], blue [465 nm] or far-red [730 nm] at a fluence rate of 35 ± 2.5 μmol m^−2^	Modulation of total phenol content and enhanced antioxidant capacity	Increased nutritive value and reduced microbiological load	[163]
Broccoli	White LED lights at 3.6 W m^−2^, 7.5 W m^−2^, 19.0 W m^−2^ intensities	Carotenoid biosynthesis, reduction of soluble sugars, and ascorbic acid degradation	Enhanced shelf life and total antioxidant profile	[161]
Citrus	Blue [455 nm] in varied photoperiods [negative control—darkness DD, 8 h light/16 h dark [8 LD], 16 h light/8 h dark [16 LD], and 24 h constant light [24 LL] at fluence rates of 50, 100, 150, and 200 μmol m^−2^ s ^−1^	Carotenoid biosynthesis, titratable acidity, and total soluble solids [at 50 μmol m^−2^ s^−1^]	Improved firmness, color, and sensory properties	[79]

## Data Availability

No new data were created or analyzed in this study.
